# Metformin attenuates the onset of non-alcoholic fatty liver disease and affects intestinal microbiota and barrier in small intestine

**DOI:** 10.1038/s41598-019-43228-0

**Published:** 2019-04-30

**Authors:** Annette Brandt, Angélica Hernández-Arriaga, Richard Kehm, Victor Sánchez, Cheng Jun Jin, Anika Nier, Anja Baumann, Amélia Camarinha-Silva, Ina Bergheim

**Affiliations:** 1 Department of Nutritional Sciences, R.F. Molecular Nutritional Science, University of Vienna, 1090 Vienna, Austria; 2 Institute of Animal Science, University of Hohenheim, 70599 Stuttgart, Germany; 3 Institute of Nutrition, SD Model Systems of Molecular Nutrition, Friedrich-Schiller-University Jena, 07743 Jena, Germany; 4 Department of Molecular Toxicology, German Institute of Human Nutrition Potsdam-Rehbruecke (DIfE), 14558 Nuthetal, Germany

**Keywords:** Non-alcoholic fatty liver disease, Nutrition disorders

## Abstract

The antidiabetic drug metformin has been proposed to affect non-alcoholic fatty liver disease (NAFLD) through its effects on intestinal microbiota and barrier function. However, so far most studies focused on long-term effects and more progressed disease stages. The aim of this study was to assess in two experimental settings, if the onset of NAFLD is associated with changes of intestinal microbiota and barrier function and to determine effects of metformin herein. C57Bl/6J mice were fed a liquid control diet (C) or fat-, fructose- and cholesterol-rich diet (FFC) for four days or six weeks ±300 mg/kg BW/day metformin (Met). Markers of liver health, intestinal barrier function and microbiota composition were assessed. Metformin treatment markedly attenuated FFC-induced NAFLD in both experiments with markers of inflammation and lipidperoxidation in livers of FFC + Met-fed mice being almost at the level of controls. Metformin treatment attenuated the loss of tight junction proteins in small intestine and the increase of bacterial endotoxin levels in portal plasma. Changes of intestinal microbiota found in FFC-fed mice were also significantly blunted in FFC + Met-fed mice. Taken together, protective effects of metformin on the onset of NAFLD are associated with changes of intestinal microbiota composition and lower translocation of bacterial endotoxins.

## Introduction

With a prevalence of approximately 25% worldwide^1^, non-alcoholic fatty liver disease (NAFLD) is thought to be the most common liver disease in the world by now. Overweight and insulin resistance have been identified as key risk factors. However, not only a general overnutrition but also an unhealthy dietary pattern e.g. an elevated intake of sugar- and fat-rich foods, a lack of physical activity and genetic predisposition as well as changes of intestinal microbiota and barrier function are discussed to be critical in the development of NAFLD (for overview see^2^). Indeed, results of several more recent studies suggest that the development of NAFLD is associated with marked changes of fecal microbiota composition and increased bacterial endotoxin levels^3–6^. Furthermore, results of animal studies and some human studies suggest that targeting intestinal microbiota and/or barrier function may have protective effects on the development of NAFLD^7–9^.

Since the early 1960s, the biguanide metformin (1,1-dimethylbiguanide) is used as an oral antidiabetic drug (for overview see^10^). While for many years it was thought that metformin exerts its beneficial effects on type 2 diabetes through mechanisms involving an AMP-activated protein kinase (AMPK)-dependent improvement of hepatic glucose metabolism and increased glucose uptake into muscle cells^11^, in more current years, studies indicate that metformin also affects intestinal microbiota composition and intestinal barrier function^12–14^. Indeed, recently in patients with type 2 diabetes effects of metformin were associated with a decreased prevalence of *Bacteroides fragilis*^15^. Furthermore, treating insulin resistant mice with this bacterial strain additionally to metformin abolished the beneficial effects of metformin on glucose tolerance^15^. Results of animal and human studies also suggest that metformin may at least in part attenuate the onset and progression of NAFLD^16–19^. Results of our own group and those of others suggest that the protective effects of metformin on the development of NAFLD are related to its effects on intestinal microbiota composition and barrier function^12,14,19,20^. However, mechanisms involved as well as if the effects of metformin on intestinal microbiota composition and barrier function are only found after an extended intake of the drug, remains to be determined.

Therefore, the present study aimed to assess if an oral treatment with metformin protects mice from the onset and progression of NAFLD. Furthermore, we determined if effects on NAFLD are associated with changes of intestinal microbiota composition and altered translocation of bacterial endotoxin.

## Results

### Metformin and the development of NAFLD: short- and long-term effects

**Figure 1:**
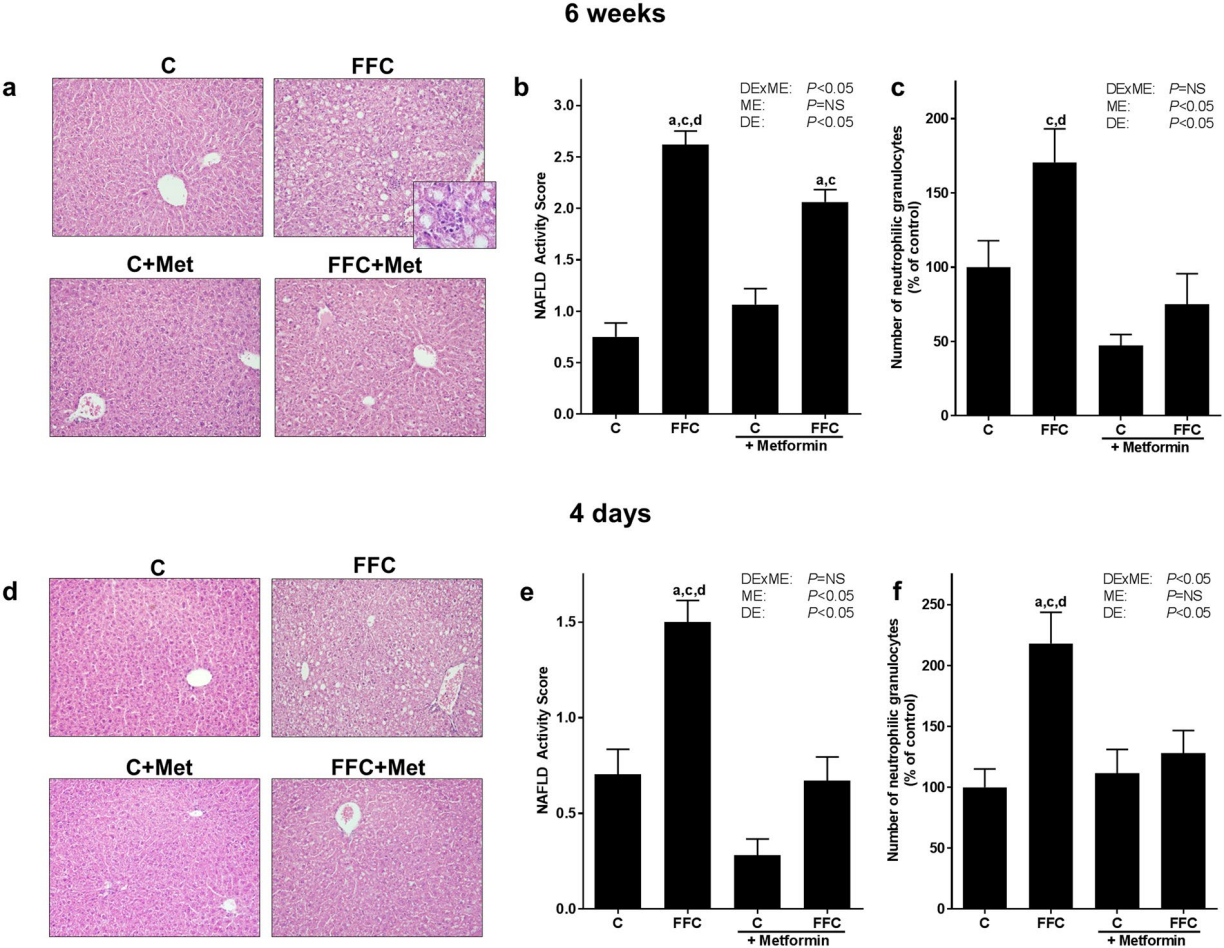
Effect of a chronic (6 weeks) or short-term (4 days) feeding of a FFC or control diet ± metformin on liver in mice. (**a**,**d**) Representative pictures of H&E staining in liver tissue (200x, 630x) and (**b,e**) evaluation of H&E staining via NAFLD Activity Score. (**c**,**f**) Number of neutrophilic granulocytes in liver tissue; n = 5–8. Data presented as means ± standard error of means. C: control diet; C + Met: control diet and oral treatment with metformin (300 mg/kg BW/day); DE: diet effect; DExME: interaction between diet and metformin; FFC: fat-, fructose- and cholesterol-rich diet; FFC + Met: fat-, fructose- and cholesterol-rich diet and oral treatment with metformin (300 mg/kg BW/day); H&E: hematoxylin and eosin; ME: metformin effect; Met: metformin; NS: not significant. ^a^*P* < 0.05 compared with mice fed a control diet; ^c^*P* < 0.05 compared with mice fed a control diet treated with metformin; ^d^*P* < 0.05 compared with mice fed a FFC diet treated with metformin.

As expected and in line with previous studies of our group^21,22^, mice fed the fat-, fructose- and cholesterol-rich diet (FFC) for six weeks developed manifest steatosis with beginning signs of inflammation (see Table 1 and Fig. 1a–c). While liver weight, liver to body weight ratio and triglyceride concentration in liver tissue were similar between FFC− and FFC + metformin (FFC + Met)-fed mice, NAFLD activity score (NAS) was significantly lower in FFC + Met-fed mice when compared with FFC-fed animals. However, NAS of FFC + Met-fed mice was still significantly higher than in livers of control (C)− and C + Met-fed mice. In line with these findings, number of neutrophilic granulocytes in liver tissue was also significantly lower in livers of FFC + Met-fed mice than in FFC-fed animals (*P* < 0.05, see Fig. 1a–c, Supplementary Fig. S1). Indeed, number of neutrophilic granulocytes did not differ between control groups and FFC + Met-fed mice. Furthermore, protein concentration of tumor necrosis factor-α (TNFα) in liver tissue was higher in FFC-fed mice than in all other groups (*P* < 0.05 for FFC vs FFC + Met, *P* = 0.06 for FFC vs C, *P* = 0.1 FFC vs C + Met, see Table 2). Expression of *interleukin 10* (*Il10*) mRNA in liver was significantly higher in FFC + Met-fed mice than in all other groups (*P* < 0.05, Supplementary Table S1), whereas *Il10* mRNA expression was almost at the level of controls in FFC-fed mice. *Interleukin 1β* (*Il1b*) and *C-type lectin domain family 10 member A (Clec10a)* mRNA expression in liver tissue was similar between groups (Table 2, Supplementary Table S1). Furthermore, in line with previous studies employing this feeding model^21^, markers of fibrosis like Sirius red staining in liver tissue, as well as hepatic mRNA expression of *α-smooth muscle actin* (*asma*) and *vimentin* did not differ between groups (Table 1, Supplementary Fig. S1). As animals only showed beginning signs of the disease, plasma alanine transaminase (ALT) activity was similar between groups. Also, fasting blood glucose levels and *phosphoenolpyruvate carboxykinase* (*Pepck*) mRNA expression in liver did not differ between groups (Table 1, Supplementary Table S1).

**Table 1:** Effect of a chronic feeding (6 weeks) of a FFC or control diet ± metformin on caloric intake, body weight gain and markers of liver damage and fasting blood glucose.

	groups				*P*-value		
	C	FFC	C + Met	FFC + Met	DExME	ME	DE
Caloric intake [kcal/mouse/d]	10.5 ± 0.1	10.8 ± 0.2	10.7 ± 0.1	10.5 ± 0.2	NS	NS	NS
Body weight [g] (at end of trial)	21.4 ± 0.6	22.1 ± 0.3	21.9 ± 0.5	22.4 ± 0.5	NS	NS	NS
Weight gain [g]	2.4 ± 0.5	2.6 ± 0.4	2.4 ± 0.4	3.0 ± 0.6	NS	NS	NS
Liver weight [g]	1.1 ± 0.0	1.4 ± 0.0^a,c^	1.1 ± 0.0	1.4 ± 0.1^a,c^	NS	NS	<0.05
Liver:Body weight ratio [%]	4.9 ± 0.1	6.4 ± 0.2^a,c^	5.0 ± 0.2	6.2 ± 0.3^a,c^	NS	NS	<0.05
Hepatic triglycerides [µg/mg protein]	11.1 ± 1.7	38.7 ± 4.9^a,c^	10.4 ± 1.6	36.5 ± 7.1^a,c^	NS	NS	<0.05
Sirius red (% of microscopic field)	1.1 ± 0.1	1.2 ± 0.1	1.1 ± 0.1	1.3 ± 0.3	NS	NS	NS
*asma* mRNA^#^	100 ± 24	79.8 ± 24	71.8 ± 14	82.9 ± 9.9	NS	NS	NS
*Vimentin* mRNA^#^	100 ± 25	126 ± 15	114 ± 26	117 ± 6.3	NS	NS	NS
Plasma ALT [U/I]	15.2 ± 1.0	18.9 ± 2.4	17.3 ± 1.2	17.7 ± 1.1	NS	NS	NS
Fasting blood glucose [mg/dL]	93 ± 6	87 ± 7	99 ± 7	95 ± 7	NS	NS	NS

Values are means ± standard error of means; n = 5–6. ^#^Values are shown as % of control. ALT: alanine aminotransferase, asma: α-smooth muscle actin, C: control diet, C + Met: control diet and oral treatment with metformin (300 mg/kg BW/day), DE: diet effect, DExME: interaction between diet and metformin, FFC: fat-, fructose- and cholesterol-rich diet, FFC + Met: fat-, fructose- and cholesterol-rich diet and oral treatment with metformin (300 mg/kg BW/day), ME: metformin effect, Met: metformin, NS: not significant. ^a^*P* < 0.05 compared with mice fed a control diet; ^c^*P* < 0.05 compared with mice fed a control diet treated with metformin.

**Table 2:** Effect of a chronic (6 weeks) and short-term feeding (4 days) of a FFC or control diet ± metformin on markers of inflammation and lipogenesis in liver tissue.

	groups				*P*-value		
	C	FFC	C + Met	FFC + Met	DExME	ME	DE
**6 weeks**							
TNFα [ng/mg protein]	0.33 ± 0.04	0.48 ± 0.06^d^	0.34 ± 0.02	0.30 ± 0.02	<0.05	<0.05	NS
*Il1b* mRNA^#^	100 ± 17	166 ± 32	114 ± 21	164 ± 25	NS	NS	<0.05
*Acc* mRNA^#^	100 ± 46	249 ± 91	82.3 ± 11	314 ± 51^a,c^	NS	NS	<0.05
*Fasn* mRNA^#^	100 ± 36	405 ± 133^a^	106 ± 21	445 ± 98^a,c^	NS	NS	<0.05
*Srebp1c* mRNA^#^	100 ± 11	395 ± 101^a,c^	132 ± 21	382 ± 56^a,c^	NS	NS	<0.05
*Acox1* mRNA^#^	100 ± 12	134 ± 26	77.8 ± 14	140 ± 17	NS	NS	<0.05
**4 days**							
TNFα [ng/mg protein]	0.20 ± 0.02	0.23 ± 0.02	0.20 ± 0.01	0.24 ± 0.01	NS	NS	<0.05
*Il1b* mRNA^#^	100 ± 20	95.0 ± 13	151 ± 35	93.4 ± 21	NS	NS	NS
*Acc* mRNA^#^	100 ± 25	111 ± 35	90.4 ± 15	128 ± 56	NS	NS	NS
*Fasn* mRNA^#^	100 ± 31	157 ± 34	71.7 ± 10	157 ± 64	NS	NS	NS
*Srebp1c* mRNA^#^	100 ± 14	216 ± 20^a,c^	115 ± 23	147 ± 13	<0.05	NS	<0.05
*Acox1* mRNA^#^	100 ± 26	165 ± 43	146 ± 20	69.1 ± 14	<0.05	NS	NS

Values are means ± standard error of means; n = 5–8. ^#^Values are shown as % of control. Acc: acetyl coA carboxylase, Acox1: acyl coA oxidase-1, C: control diet, C + Met: control diet and oral treatment with metformin (300 mg/kg BW/day), DE: diet effect, DExME: interaction between diet and metformin, Fasn: fatty acid synthase, FFC: fat-, fructose- and cholesterol-rich diet, FFC + Met: fat-, fructose- and cholesterol-rich diet and oral treatment with metformin (300 mg/kg BW/day), ME: metformin effect, Met: metformin, NS: not significant, Il1b: interleukin 1β, Srebp1c: sterol regulatory element-binding protein 1, TNFα: tumor necrosis factor-α. ^a^*P* < 0.05 compared with mice fed a control diet; ^c^*P* < 0.05 compared with mice fed a control diet treated with metformin; ^d^*P* < 0.05 compared with mice fed a FFC diet treated with metformin.

Despite only having been fed the FFC for four days, mice also had developed beginning signs of NAFLD with significantly higher NAS, hepatic triglyceride concentration and number of neutrophilic granulocytes as well as F4/80 positive cells in livers compared to C-fed mice (*P* < 0.05, see Fig. 1d–f, Table 3, Supplementary Fig. S1). As mice show only very early signs of NAFLD, liver weight, liver to body weight ratio and ALT activity in plasma did not differ between FFC-fed groups. Still, metformin treatment attenuated these beginning signs of NAFLD with NAS, hepatic triglyceride concentration and number of neutrophils all being significantly lower than in the group only fed FFC. However, triglyceride levels in liver tissue of FFC + Met-fed mice were still significantly higher than in controls whereas NAS, number of neutrophils and F4/80 positive cells did not differ from C- and C + Met-fed mice (Fig. 1d–f, Table 3 and Supplementary Fig. S1). Expression of pro- and anti-inflammatory markers like *Il10*, *Il1b* and *Clec10a* as well as protein concentration of TNFα in liver tissue were similar between groups (Table 2, Supplementary Table S1). Furthermore, *Pepck* mRNA expression in liver was significantly lower in both FFC-fed groups compared to C + Met-fed mice (*P* < 0.05, Supplementary Table S1).

**Table 3:** Effect of a short-term feeding (4 days) of a FFC or control diet ± metformin on caloric intake, body weight gain, markers of liver damage.

	groups				*P*-value		
	C	FFC	C + Met	FFC + Met	DExME	ME	DE
Caloric intake [kcal/mouse/d]	10.3 ± 0.4	10.6 ± 0.3	10.1 ± 0.2	10.3 ± 0.6	NS	NS	NS
Body weight [g] (at end of trial)	19.0 ± 0.5	20.0 ± 0.4	20.1 ± 0.4	19.9 ± 0.5	NS	NS	NS
Weight gain [g]	1.0 ± 0.5	1.7 ± 0.2	1.6 ± 0.3	1.3 ± 0.8	NS	NS	NS
Liver weight [g]	1.0 ± 0.1	1.2 ± 0.1	1.0 ± 0.0	1.2 ± 0.1	NS	NS	<0.05
Liver:Body weight ratio [%]	5.4 ± 0.3	6.1 ± 0.2^c^	5.2 ± 0.1	6.1 ± 0.3^c^	NS	NS	<0.05
Hepatic triglycerides [µg/mg protein]	13.1 ± 2.5	54.3 ± 5.6^a,c,d^	17.9 ± 2.8	31.5 ± 3.9^a^	<0.05	<0,05	<0.05
Plasma ALT [U/I]	17.1 ± 2.1	18.2 ± 1.8	14.7 ± 1.5	13.0 ± 1.8	NS	<0.05	NS
F4/80 positive cells (number/microsc. field)	20.6 ± 1.1	25.0 ± 0.7^a,c^	20.9 ± 0.8	21.5 ± 1.2	NS	NS	<0.05

Values are means ± standard error of means; n = 7–8. ALT: alanine aminotransferase, C: control diet, C + Met: control diet and oral treatment with metformin (300 mg/kg BW/day), DE: diet effect, DExME: interaction between diet and metformin, FFC: fat-, fructose- and cholesterol-rich diet, FFC + Met: fat-, fructose- and cholesterol-rich diet and oral treatment with metformin (300 mg/kg BW/day), ME: metformin effect, Met: metformin, NS: not significant. ^a^*P* < 0.05 compared with mice fed a control diet; ^c^*P* < 0.05 compared with mice fed a control diet treated with metformin;. ^d^*P* < 0.05 compared with mice fed a FFC diet treated with metformin.

### Metformin and NAFLD: hepatic lipid metabolism

After six weeks of feeding, hepatic mRNA expressions of *sterol regulatory element-binding protein 1* (*Srebp1c*) and *fatty acid synthase* (*Fasn*) were markedly higher in both FFC-fed groups compared to both control groups (Table 2, Srebp1c: *P* < 0.05 for FFC and FFC + Met vs C and C + Met, Fasn: *P* < 0.05 for FFC and FFC + Met vs C; for FFC + Met vs C + Met), while mRNA expression of *acetyl coA carboxylase* (*Acc)* was only significantly higher in livers of FFC + Met-fed mice than in both control groups. Expression of *acyl coA oxidase-1* (*Acox1*) mRNA was similar between groups (Table 2).

After four days of treatment neither expression of *Acc* nor *Fasn* or *Acox1* mRNA differ between groups (Table 2). However, *Srebp1c* mRNA expression was significantly induced in livers of FFC-fed mice compared to both control groups (*P* < 0.05, Table 2) whereas in livers of FFC + Met-fed mice *Srebp1c* mRNA expression levels were almost at the level of controls (Table 2).

### Metformin and NAFLD: TLR4 and dependent signaling cascades in liver

**Figure 2:**
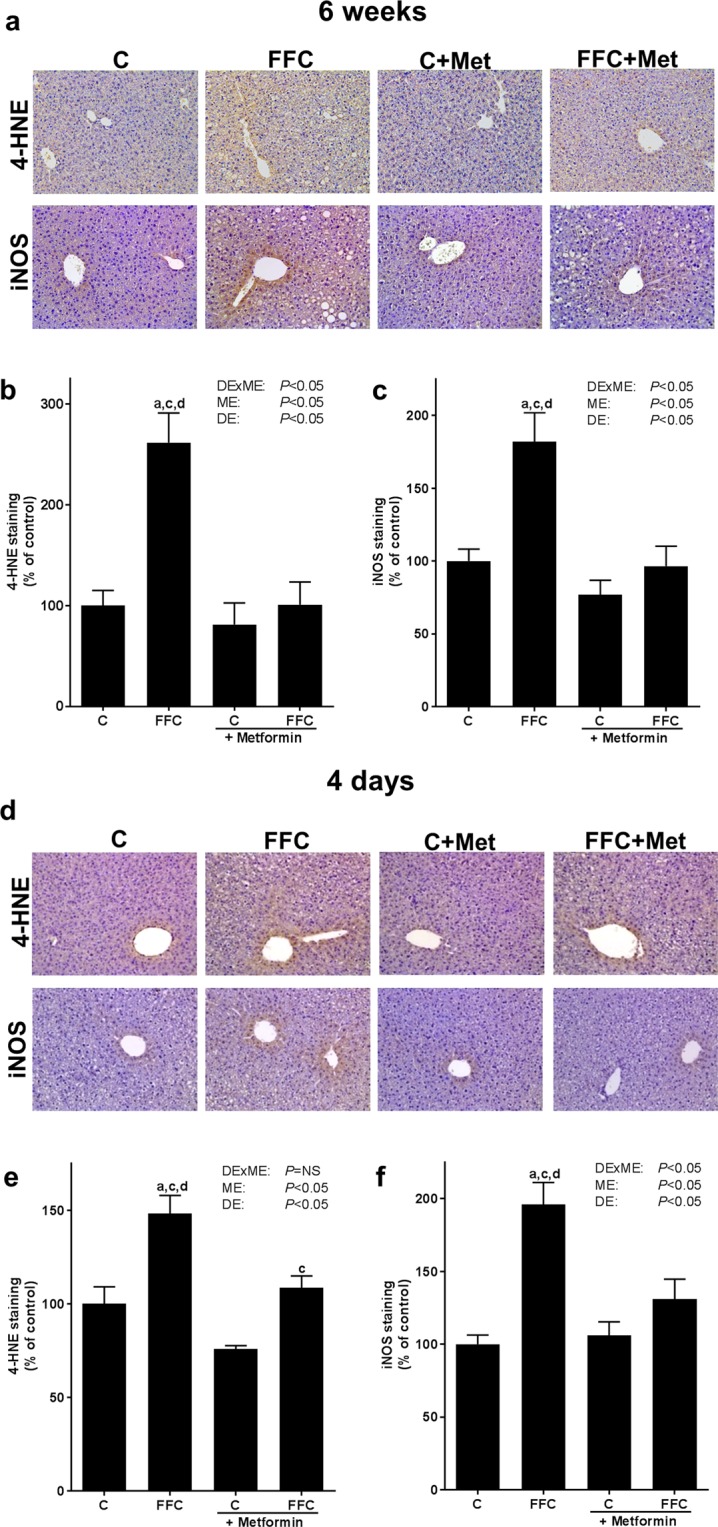
Effect of a chronic (6 weeks) or short-term (4 days) feeding of a FFC or control diet ± metformin on inflammation and markers of lipidperoxidation in mice. (**a**,**d**) Representative pictures (200x) and densitometric analysis of (**b**,**e**) 4-HNE protein adducts and (**c**,**f**) iNOS protein staining in hepatic tissue; n = 6–8. Data presented as means ± standard error of means. 4-HNE: 4-hydroxynonenal; C: control diet; C + Met: control diet and oral treatment with metformin (300 mg/kg BW/day); DE: diet effect; DExME: interaction between diet and metformin; FFC: fat-, fructose- and cholesterol-rich diet; FFC + Met: fat-, fructose- and cholesterol-rich diet and oral treatment with metformin (300 mg/kg BW/day); iNOS: inducible nitric oxide synthase; ME: metformin effect; Met: metformin; NS: not significant. ^a^*P* < 0.05 compared with mice fed a control diet; ^c^*P* < 0.05 compared with mice fed a control diet treated with metformin; ^d^*P* < 0.05 compared with mice fed a FFC diet treated with metformin.

In FFC-fed groups, hepatic *toll-like receptor 4 (Tlr4)* mRNA expression was significantly higher than in C + Met-fed mice, whereas similar differences were not found when comparing FFC-fed groups and C-fed mice after six weeks of feeding (see Table 4). In contrast, myeloid differentiation primary response 88 (MyD88) protein concentration in liver tissue was significantly higher in FFC-fed mice compared to all other groups (*P* < 0.05 for FFC vs C, C + Met and FFC + Met, see Table 4 and Supplementary Fig. S1). Phosphorylation of inhibitor of nuclear factor kappa-B kinase subunit α and β (pIKKα/β) was higher in FFC-fed mice compared to all other groups; however, as data varied considerable within groups differences didn´t reach the level of significance (Supplementary Fig. S2). Protein concentrations of inducible nitric oxide synthase (iNOS) and 4-hydroxynonenal protein adducts (4-HNE) in hepatic tissue of FFC-fed mice were also significantly higher compared to all other groups while concentrations of both markers were at the level of controls in livers of FFC + Met-fed mice (see Fig. 2a–c).

**Table 4:** Effect of a chronic (6 weeks) and short-term feeding (4 days) of a FFC or control diet ± metformin on mRNA expression of tight junction proteins in proximal small intestine and markers of TLR4 signaling cascade in liver tissue.

	groups				*P*-value		
	C	FFC	C + Met	FFC + Met	DExME	ME	DE
**6 weeks**							
*Tlr4* mRNA	100 ± 15	121 ± 12^c^	71.4 ± 5.6	132 ± 8.0^c^	NS	NS	<0.05
MyD88 protein staining	100 ± 21	177 ± 20^a,c,d^	58.1 ± 14	86.8 ± 19	NS	<0.05	<0.05
*Occ mRNA*	100 ± 23	87.9 ± 36	93.1 ± 37	81.5 ± 25	NS	NS	NS
**4 days**							
*Tlr4* mRNA	100 ± 15	107 ± 10	129 ± 15	96.8 ± 9.4	NS	NS	NS
MyD88 protein staining	100 ± 8.1	215 ± 15^a^	154 ± 13^a^	168 ± 25^a^	<0.05	NS	<0.05
*Occ mRNA*	100 ± 31	169 ± 76	105 ± 22	118 ± 37	NS	NS	NS
*Zo1 mRNA*	100 ± 28	97.2 ± 31	76.3 ± 10	83.3 ± 26	NS	NS	NS

Values are means ± standard error of means and are shown as % of control; n = 4–8. C: control diet, C + Met: control diet and oral treatment with metformin (300 mg/kg BW/day), DE: diet effect, DExME: interaction between diet and metformin, FFC: fat-, fructose- and cholesterol-rich diet, FFC + Met: fat-, fructose- and cholesterol-rich diet and oral treatment with metformin (300 mg/kg BW/day), ME: metformin effect, Met: metformin, MyD88: myeloid differentiation primary response 88, NS: not significant, Occ: occludin, Tlr4: toll like receptor 4, Zo1: zonula occludens-1. ^a^*P* < 0.05 compared with mice fed a control diet; ^c^*P* < 0.05 compared with mice fed a control diet treated with metformin;. ^d^*P* < 0.05 compared with mice fed a FFC diet treated with metformin.

After four days of feeding the FFC, expression of *Tlr4* mRNA in liver tissue did not differ between groups. Protein levels of MyD88 protein were significantly higher in FFC−, FFC + Met- and C + Met-fed mice than in C-fed mice. MyD88 protein levels did not differ between FFC-fed groups (see Table 4 and Supplementary Fig. S1). In line with the findings in mice fed the FFC for six weeks, concentrations of 4-HNE protein adducts and iNOS protein in liver tissue were both significantly higher in mice fed the FFC for only four days than in all other groups (*P* < 0.05, see Fig. 2d–f). In livers of FFC + Met-fed mice these parameters were at the level of C-fed mice; however, 4-HNE protein adduct concentration in liver tissue of FFC + Met-fed mice was still significantly higher than in livers of C + Met-fed mice (*P* < 0.05, see Fig. 2d–f).

### Metformin and NAFLD: tight junction proteins and MMP13 in proximal small intestine

**Figure 3:**
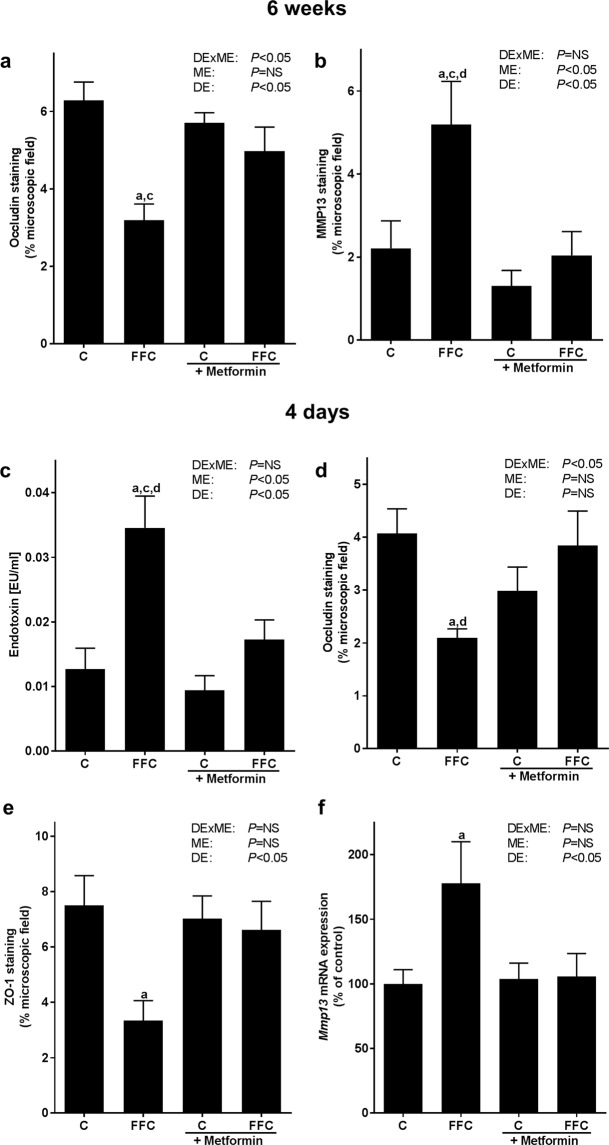
Effect of a chronic (6 weeks) or short-term feeding (4 days) of a FFC or control diet ± metformin on markers of intestinal permeability and MMP13 in mice. Densitometric analysis of (**a**) occludin protein and (**b**) MMP13 protein staining in proximal small intestine in long-term trial. (**c**) Endotoxin concentration in plasma of portal vein and densitometric analysis of (**d**) occludin and (**e**) ZO-1 protein staining in tissue of proximal small intestine, as well as (**f**) *Mmp13* mRNA expression in proximal small intestine; n = 4–8. Data presented as means ± standard error of means. C: control diet; C + Met: control diet and oral treatment with metformin (300 mg/kg BW/day); DE: diet effect; DExME: interaction between diet and metformin; FFC: fat-, fructose- and cholesterol-rich diet; FFC + Met: fat-, fructose- and cholesterol-rich diet and oral treatment with metformin (300 mg/kg BW/day); ME: metformin effect; MMP13: matrix-metalloproteinase 13; NS: not significant; ZO-1: zonula occludens-1. ^a^*P* < 0.05 compared with mice fed a control diet; ^c^*P* < 0.05 compared with mice fed a control diet treated with metformin; ^d^*P* < 0.05 compared with mice fed a FFC diet treated with metformin.

After six weeks of feeding, protein levels of the tight junction protein occludin in proximal small intestine were significantly lower in FFC-fed than in C-fed mice. A similar decrease in occludin protein concentration was not found in FFC + Met-fed mice with protein levels being almost at the level of controls (*P* = 0.05 for FFC vs FFC + Met, see Figs 3a and S3). In line with previous findings of our group^23^, mRNA expression of *occludin* in proximal small intestine was similar between groups (Table 4). Protein concentrations of matrix-metalloproteinase 13 (MMP13), a matrix-metalloproteinase suggested to be involved in degradation of tight junction proteins^24,25^, were significantly higher in proximal small intestine of FFC-fed mice than in all other groups while MMP13 protein concentrations in proximal small intestine of FFC + Met-fed animals were almost at the level of controls (see Figs 3b and S3).

Bacterial endotoxin levels in portal plasma of mice fed the FFC for only four days were significantly higher than in all other groups. Protein concentrations of occludin and zonula occludens-1 (ZO-1) were significantly lower in proximal small intestine of FFC-fed mice when compared to C-fed animals (*P* < 0.05; Figs 3c–e and S3), while neither occludin nor ZO-1 protein levels in proximal small intestine differed between FFC + Met-fed mice and C− or C + Met-fed animals. Indeed, occludin protein levels in proximal small intestine were even significantly lower in FFC-fed mice than in FFC + Met-fed animals. In line with the findings in animals fed the diets for six weeks, mRNA expression of *occludin* and *Zo1* mRNA in proximal small intestine did not differ between groups (Table 4). Also, in distal small intestine, no differences in protein concentration of both tight junction proteins were found (see Supplementary Figs S3 and S4). *Mmp13* mRNA expression was significantly higher in proximal small intestine of FFC-fed mice than in C-fed, while *Mmp13* expression was similar between C−, C + Met- and FFC + Met-fed mice (see Fig. 3f).

### Metformin and NAFLD: intestinal microbiota

**Figure 4:**
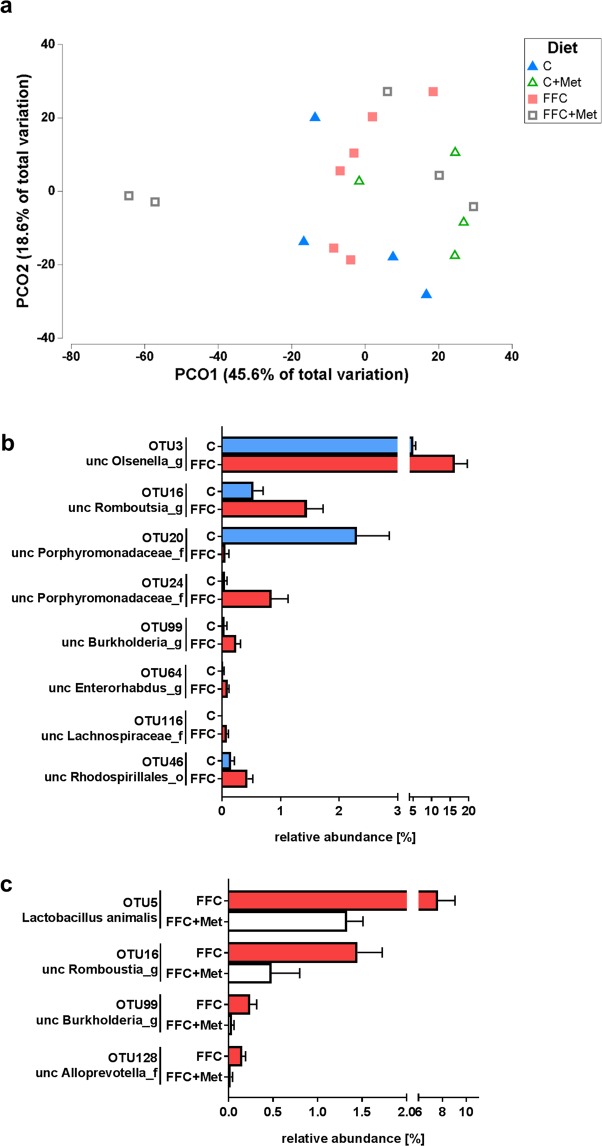
Effect of a short-term feeding (4 days) of a FFC or control diet ± metformin on microbial community in small intestine at species level. (**a**) PCO plot showing the microbial communities of each sample and relative abundance of OTU with significant difference between (**b**) control and FFC-fed mice or (**c**) FFC and FFC + Met-fed mice; n = 4–6. Data presented as means ± standard error of means. C: control diet; C + Met: control diet and oral treatment with metformin (300 mg/kg BW/day); FFC: fat-, fructose- and cholesterol-rich diet; FFC + Met: fat-, fructose- and cholesterol-rich diet and oral treatment with metformin (300 mg/kg BW/day); PCO: principal coordinate analysis; unc: unclassified.

To further determine if the changes found in markers of intestinal barrier function and the protective effects found in FFC-fed mice treated with metformin were associated with changes of intestinal microbiota composition in small intestine, 16S rRNA gene amplicon sequencing was performed in proximal small intestine of mice fed the different diets for four days. ANOSIM analysis indicated that the intestinal microbial communities differed significantly between all groups (*P* = 0.003). Principal Coordinate Analysis (PCoA) further revealed a clustering of bacterial structure between the different feeding and treatment groups along the primary ordinate axid (OC1) which accounted for ~47.3% of the total variation (see Fig. 4a). A total of 92 operative taxonomic units (OTU) were shared by all groups (see Supplementary Fig. S5). The average similarity among replicates within the groups was 54% in C-fed mice, 65% in FFC-fed mice, 67% in C + Met-fed mice and 32% in FFC + Met-fed mice. Neither Shannon’s Diversity Index nor Pillow´s Evenness differed among groups, probably as relative abundance of some OTUs was rather low (<0.5%) (data not shown). Differences in small intestinal microbiota were further compared between selected groups. Compared to C-fed mice relative abundance of OTU level of unclassified members of the genera *Burkholderia*, *Enterohabdus*, *Olsenella* and *Romboutsia* as well as some unclassified members of the Lachnospiraceae family and the Rhodospirillales order were significantly higher in small intestine of FFC-fed mice (see Fig. 4b). Two OTUs assigned to unclassified Porphyromonadaceae were either more abundant in the C-fed mice or in FFC-fed mice. When comparing relative abundance at species level in small intestine between FFC- and FFC + Met-fed mice, relative abundance of the species *Lactobacillus animalis*, unclassified members of *Burkholderia* and *Romboutsia* genera and an unclassified member of Alloprevotella family were significantly lower in small intestine of FFC + Met-fed mice compared to FFC-fed animals (see Fig. 4c). No differences were found in relative abundance of *Akkermansia muciniphila* or *Bacteroides* genus between C− and FFC− or FFC- and FFC + Met-fed mice.

## Discussion

Metformin is one of the first line medications in the management of type 2 diabetes (for overview also see^26^). Results from animal studies further suggest that it may also be a therapy of NAFLD with rather marked effects (for overview see^26^); however, data from human trials are contradictory and less promising^27^. Still, as there is so far no universally accepted treatment for the management of NAFLD available, a better understanding of the beneficial effects of metformin found in animal studies may add to improve its effects in the human situation. In line with the findings of others in animal models^17,28,29^, in the present study, oral treatment with metformin attenuated at least in part the development of both, the early onset of fatty liver and the progression of NAFLD to steatosis accompanied with inflammation. However, in line with previous studies of us and others^21,30^, markers of fibrosis were not yet altered in any treatment group as trials were terminated already after six weeks. Metformin markedly protected FFC-fed animals from fat accumulation in liver and the slight increase in number of pro-inflammatory cells after four days, while after six weeks, fat accumulation was almost similar between FFC-fed groups. In line with the findings that metformin treatment protected mice from hepatic fat accumulation when mice were fed the FFC diet for only four days, the induction of *Srebp1c* mRNA expression found in FFC-fed mice was markedly attenuated in FFC + Met-fed mice. Indeed, metformin has been reported before to diminish de novo lipogenesis by inhibiting proteolytic cleavage and transcriptional activity of SREBP-1c in hepatocytes treated with glucose and insulin^31^. Expression of genes involved in β-oxidation seemed not to have been affected by either the FFC diet or metformin in the present study. In support of these findings, other groups also found genes involved in β-oxidation to be unchanged in a dietary model of NAFLD after eight weeks of feeding while with the same diet, markers of β-oxidation were found to be markedly changed after 16 weeks of feeding when mice had developed NASH^32^. Together with the data of the present study these data suggest that alterations of genes involved in β-oxidation in liver maybe a phenomena of later stages of the disease. In line with the lack of effect of metformin on fat accumulation after six weeks of treatment, expression of genes involved in hepatic de novo lipogenesis e.g. *Acc*, *Fasn*, *Srebp1c* were induced to a similar extend in both FFC-fed groups. Still, after six weeks of feeding signs of inflammation in liver were lower in FFC + Met-fed mice being associated with an induction of *Il10* mRNA expression in hepatic tissue. In *in vitro* experiments in LPS-stimulated macrophages metformin treatment has been shown to induce *Il10* mRNA expression before^33^. ALT activities in plasma were not affected by diet and/or metformin treatment at any time point. However, studies suggest that adipose tissue insulin resistance and low circulating adiponectin levels as well as high hepatic triglyceride levels may be a major factor in the elevation of plasma transaminase activity^34^. In the present study, a pair-feeding model was employed, so even after six weeks of feeding FFC-fed mice stayed normal weight. Furthermore, studies of others suggest that in normal weight rodents fed a high-fat diet, protein to carbohydrate ratio seems to be critical in impairing glucose homeostasis^35^. Indeed, similar to the findings in the present study, in these studies^35^ fasting glucose levels remain unaltered further suggesting that dietary composition and body weight may be critical determinants of transaminase activity and glucose homeostasis. Furthermore, *Pepck* mRNA expression, a key enzyme of gluconeogenesis shown before to be induced in livers of rodents fed a high-fat high-fructose diet^36^ did not differ between groups after six weeks of treatment. Differences between the findings of the present study and those of others may have resulted from differences in experimental design as well as the lack of massive body weight gain of animals in the present study. Also, in *in vitro* studies Foretz *et al*. showed that metformin may alter expression of the catalytic subunit of glucose-6-phosphate (G6pase), while in this study *Pepck* expression was unaltered^37^. However, when mice were fed the FFC diet for only four days, *Pepck* mRNA expression was markedly lower in both FFC-fed mouse groups compared to controls further suggesting that expression of *Pepck* may be adapted to the availability of certain macronutrients. It has been demonstrated in *in vitro* studies that free fatty acids may exert short-term inhibitory effects on gluconeogenic response through lowering *Pepck* and *G6pase* expression^38^.

Taken together, our data suggest that in the present study employing a fat-, fructose-, and cholesterol-rich pair-fed normo-caloric diet, metformin treatment ‘delayed´ the development of NAFLD rather than attenuating it completely. However, as in the present study interventions were terminated after four days and six weeks, respectively, it cannot be precluded that beneficial effects of metformin maybe abolished when treatment is extended. This needs to be addressed in future studies.

Several human and animal studies suggest that alterations of intestinal microbiota composition, barrier function and subsequently elevated bacterial endotoxin levels are critical in the development of NAFLD (for overview^2^). Furthermore, in settings of type 2 diabetes, the beneficial effects of metformin have been suggested to be related to intestinal microbiota composition^13,15^ and barrier function^14^. In the present study, the protection against the onset and the more progressed stages of NAFLD found in metformin treated animals were associated with an attenuation of the loss of tight junction proteins in proximal small intestine but not in distal small intestine, and of the induction of MMP13 in proximal small intestine, the latter suggested to be critical in the degradation of tight junction proteins^24,25^. In line with previous findings of our group^23^, mRNA expressions of tight junction proteins were neither altered after the short nor the long-term intervention. Somewhat contrasts the findings of others reporting that the protective effects of metformin on the development of NAFLD were associated with a protection from the loss of occludin and ZO-1 in ileum and colon^14^, in the present study effects on tight junction proteins were found in proximal small intestine. However, experimental set-up markedly differed between our study and that of Zhou *et al*.^14^ Still, in line with the present study and our earlier findings^19^, Zhou *et al*. also showed that oral treatment with metformin abolished the translocation of endotoxin into the portal system^14^ subsequently leading to a protection against induction of TLR4-dependent signaling cascades in liver^19^. It has been shown by us and others, that the intake of a fat- and fructose-rich diet or high-fat diet in rodents can result in a marked increase in portal endotoxin levels and induction of depending signaling cascade in the liver within days^7,39^. Furthermore, while in the present study *Tlr4* mRNA expression in liver tissue was unchanged (four days) or only slightly higher in both FFC-fed groups when compared to C + Met-fed mice after six weeks of feeding, MyD88 protein levels in liver were only found to be significantly higher in mice fed the FFC for six weeks. It has been suggested before that the presence of elevated bacterial endotoxin can alter expression of *Tlr4* mRNA in liver tissue^5,40^; however, it has also been suggested that the abundance of *Tlr4* mRNA in liver tissue doesn´t always reflect the presence of bacterial endotoxin as the receptor is not solely regulated at the level of transcription^41^. Furthermore, FFC + Met-fed mice were significantly protected against the increase in iNOS protein and 4-HNE protein adduct concentration found in livers of FFC-fed mice. Also, while not reaching the level of significance, phosphorylation of IKKα/β shown to be upstream in the regulation of NFκB, was also lower in livers of FFC + Met-fed mice when compared to FFC-fed animals. The induction of iNOS in liver tissue has been shown to be highly dependent upon the presence of bacterial endotoxin and an activation of NFκB in settings of NAFLD^7,19,42^. Taken together, our data lend further support to the hypothesis that the beneficial effects of an oral treatment with metformin on the development of NAFLD may at least in part result from a protection against impairments of intestinal barrier function e.g. the loss of tight junction proteins and subsequently an increased translocation of bacterial endotoxin. Our data also suggest that these changes are already found within days. However, our data by no means preclude that metformin may also activate AMPK in liver and maybe also in other tissues thereby furthering its beneficial effects.

It is by now well established that diet affects composition of intestinal microbiota and that for instance shifting dietary pattern from a low-fat/plant polysaccharide-rich diet towards a high-fat/high-sugar diet may alter intestinal microbiota composition in rodents and humans within days^43,44^. Metformin has been shown to impact members of the intestinal microbiota in both healthy as well as disease states^15,45^ and that the beneficial effects of metformin on glucose metabolism may result from changes in the abundance of *Akkermansia muciniphila*^12,20,46^ and *Bacteriodes fragilis*^15^, respectively. In line with these findings, in the present study, both, exchanging the liquid control diet with the FFC and adding metformin to these diets were associated with a marked change in microbial communities present in proximal small intestine and relative abundance of certain bacterial strains already after four days. However, relative abundance of *Akkermansia muciniphila* in proximal small intestine was not altered between C−, FFC− and FFC + Met-fed mice in the present study. Differences between the results of others and our study might have resulted from different study design and the intestinal section studied^12,20^. Indeed, it has been suggested that fecal microbiome may markedly differ from colon mucosa microbiome and even more so small intestinal and colon microbiome^47,48^. Furthermore, we detected no differences for the genus *Bacteroides* between C−, FFC− and FFC + Met-fed mice and at species level *Bacteroides fragilis* was not identified, so it cannot be ruled out that relative abundance of *Bacteriodes fragilis* was altered in FFC + Met-fed animals. Still, data of the present study suggest that the protective effects found in FFC + Met-fed mice were not associated with an increase in the abundance of a particular bacterial strain but rather that relative abundance of certain bacterial strains and herein especially of *Burkholderia* and *Romboutsia* found to be significantly higher in FFC-fed mice when compared to C-fed mice and remained unaltered in small intestine of FFC + Met-fed animals. Indeed, it has been suggested that species of *Burkholderia* genus *Burkholderia pseudomallei* may be critical in the development of hepatocellular damage and inflammatory alterations^49^, while a decrease in the prevalence of *Romboutsia* has recently been associated with a protection against the development of high-fat diet-induced fatty liver^50^. However, if these bacterial genera are causally involved in the development of NAFLD and if the beneficial effects found in the present study are related to their decreased relative abundance as well as molecular mechanisms involved remains to be determined.

Taken together, results of the present study suggest that metformin partially attenuated the development of NALFD and that this is related to a protection against the loss of tight junction proteins and increased portal endotoxin level. Furthermore, in the present study oral treatment with metformin changed intestinal microbiota composition in proximal small intestine within days. However, our results also suggest that an increased abundance of *Akkermansia muciniphila* was not associated with the beneficial effects of metformin on the development of steatosis found in the present study. Rather, our data suggest that metformin may attenuate some of the changes induced through feeding a fat-, fructose- and cholesterol-rich diet in small intestine and liver. If these changes or an increase of a specific bacterial strain are responsible for the effects of metformin on markers of intestinal barrier function remains to be determined.

## Methods

### Animals and treatment

For all animal experiments female C57BL/6J mice (6–8 weeks old) (Janvier SAS, Le Genest-Saint-Isle, France) were housed in a specific-pathogen-free barrier facility accredited by the Association for Assessment and Accreditation of Laboratory Animal Care. All procedures were approved by the local institutional animal care and use committee (“Regierungspräsidium Stuttgart” and “Landesamt für Verbraucherschutz, Thuringia) and animals were handled in accordance to the European Convention for the Protection of Vertebrate Animals used for Experimental and Other Scientific Purposes. All experiments were carried out under controlled conditions and mice had free access to tap water at all times. Long-term experiments: As detailed previously^51^, mice had to be adapted to the liquid control diet (C; 69E% carbohydrates, 12E% fat, 19E% protein; Ssniff, Soest, Germany). Animals (n = 6/group) were then randomly assigned to the following groups: mice receiving a fat-, fructose- and cholesterol-rich diet (FFC; 60E% carbohydrates, 25E% fat, 15E% protein with 50% wt/wt fructose and 0.16% wt/wt cholesterol; Ssniff, Soest, Germany), mice receiving the FFC enriched with the oral insulin sensitizer metformin (FFC + Met, 300 mg/kg BW/day metformin; Sigma Aldrich, Steinheim, Germany;^19^), mice receiving C diet or mice receiving C diet enriched with metformin (C + Met, 300 mg/kg BW/day metformin). For six weeks animals were pair-fed these diets with caloric intake being adjusted daily to the group with the lowest caloric intake. Body weight was assessed weekly and metformin treatment was adjusted accordingly. In week four of the feeding trial mice were fasted for 6 h and blood was taken from retrobulbar venous plexus under isoflurane anesthesia. Fasting blood glucose levels were assessed with a glucometer (Bayer Consumer Care AG, Basel, Switzerland). Short-term experiments: As detailed above mice were first adapted to the consumption of the liquid control diet. Four days prior to starting the feeding of the different diets, some mice (n = 6–8/group) were fed a liquid control diet enriched with metformin (300 mg/kg BW/day). In line with the experimental set-up detailed above, mice were then assigned to the following groups for another four days: control diet (C), control diet enriched with metformin (C + Met), fat-, fructose- and cholesterol-rich diet (FFC) and FFC enriched with metformin (FFC + Met). Again, caloric intake was adjusted daily between groups. Experimental set-ups are shown in Supplementary Fig. S6. Composition of diets is summarized in Supplementary Table S2. At the end of the trials mice were anesthetized with a ketamine/xylazine mixture (100 mg ketamine/kg BW; 16 mg xylazine/kg BW) through intraperitoneal injection. Blood was obtained from portal vein just before killing and liver as well as intestinal tissue samples were collected and snap-frozen or fixed in neutral-buffered formalin.

### Histological evaluation of liver sections and hepatic lipid accumulation

Paraffin-embedded liver sections (5 µm) were stained with hematoxylin and eosin (Sigma-Aldrich, Steinheim, Germany) to evaluate status of liver damage using the NAFLD Activity Score (NAS) including steatosis, inflammatory alterations and ballooning^52^. Numbers of neutrophilic granulocytes and F4/80 positive cells in liver sections were assessed using the commercially available Naphthol AS-D Chloroacetate kit (Sigma-Aldrich, Steinheim, Germany) and immunohistochemical staining as described previously^19,53^. To determine triglyceride concentration in liver homogenates triglycerides were extracted and measured as previously described^19^. Picrosirius red (Sigma-Aldrich, Steinheim, Germany) staining was used to determine collagen deposition in hepatic sections as previously described^21^.

### Blood parameters of liver damage, endotoxin measurement and ELISA

Alanine transaminase (ALT) activity in portal plasma of mice was determined in a routine laboratory (University Hospital of Jena, Jena, Germany). Furthermore, bacterial endotoxin levels in portal plasma were measured using a limulus amebocyte lysate assay (Charles River, Ecully, France) as detailed previously^54^. A commercially available TNFα ELISA (Assaypro, St. Charles, MO, USA) was used to determine TNFα protein concentration in liver homogenate of mice.

### Immunohistochemical staining of liver and intestinal tissue

Immunohistochemical staining methods were used to measure concentration of 4-HNE, iNOS and MyD88 in paraffin embedded liver sections as well as of MMP13, occludin and ZO-1 in small intestinal tissue sections as previously described^7,19,22^. In brief, after treating sections with citrate buffer (MMP13) or incubating with protease (occludin, ZO-1) and blocking tissue sections with bovine serum albumin solution (iNOS, MMP13, MyD88), they were incubated with specific primary antibodies (4-HNE: AG Scientific, San Diego, CA, USA; iNOS: Thermo Fisher Scientific, Waltham, MA, USA; MMP13: LifeSpan BioSciences, Seattle, WA, USA; MyD88: Santa Cruz Biotechnology, Dallas, TX, USA; Occludin and ZO-1: Invitrogen, Carlsbad, CA, USA). Subsequently, sections were incubated with peroxidase-linked secondary antibodies followed by diaminobenzidine (Peroxidase Envision Kit, DAKO, Hamburg, Germany). Staining was evaluated using a camera integrated in a microscope (Leica DM4000 B LED, Leica, Wetzlar, Germany) and an analysis system (Leica Applications Suite, Leica, Wetzlar, Germany) as described previously^19,42^.

### RNA isolation and real-time RT-PCR

To determine mRNA expression in liver and intestinal tissue of mice, RNA was extracted from proximal small intestinal and liver tissue using peqGOLD Trifast (Peqlab, Erlangen, Germany) and cDNA was synthetized (Reverse Transcription System, Promega GmbH, Madison, WI, USA) as detailed elsewhere^19^. Real-time polymerase chain reaction (PCR) was used to determine expression of the respective genes as detailed before^51^. Primer sequences are shown in Supplementary Table S3.

### Western blot

To determine protein levels of IKKβ and pIKKα/β Western blot analysis were performed as previously described^42^. In brief, cytosolic protein from liver tissue was isolated using a lysis buffer (1 M HEPES, 1 M MgCl_2_, 2 M KCl and 1 M DTT) supplemented with protease and phosphatase inhibitors (Sigma-Aldrich, Steinheim, Germany). After separating protein lysates in a sodium dodecyl sulphate-polyacrylamide gel and transferring them to a Hybond^TM^-P polyvinylidene difluoride membrane, membranes were incubated with specific primary antibodies (IKKβ, pIKKα/β, both Cell Signaling Technology, Danvers, MA, USA) and respective secondary antibodies. Super Signal Western Dura kit (Thermo Fisher Scientific, Waltham, MA, USA) was used to detect protein bands and densitometric analysis were performed using ChemiDoc XRS System.

### Illumina amplicon sequencing of microbiota from small intestinal content

Bacterial DNA was extracted from proximal small intestine of 4–6 mice per group using Fast DNA SPIN Kit for Soil (MP Biomedicals, Solon, OH, USA) following the manufacturer’s instructions. Illumina Amplicon Sequencing libraries were prepared with the extracted DNA, as previously described by Camarinha-Silva *et al*.^55^, amplifying the V1-2 region of the 16S rRNA gene using the primers 27Fmod and 338R^56^. A two-step PCR using Taq primer star HS DNA (Clontech Laboratories, Mountain View, CA, USA) with an annealing temperature of 55 °C, using 15 cycles for the first PCR and 20 cycles for the second was performed. No negative control corrections were applied as PCR products were discarded if there was any evidence of product formation in the negative control when being checked on an agarose gel. Amplicons were purified and normalized using the SequalPrep^TM^ Normalization Plate Kit (Thermo Fisher Scientific, Waltham, MA, USA). Samples were sequenced on an Illumina MiSeq platform using 250 bp paired-end sequencing chemistry. Raw reads were quality filtered, assembled and aligned using Mothur pipeline^57^. After quality filtering, a total of 523,785 reads were obtained with an average of 27,568 ± 19,682 reads per sample. Reads were clustered at 97% identity into 697 operative taxonomic units (OTU). Only OTUs present at an average abundance higher than 0.0001% and a sequence length >250 bp were considered for further analysis. The closest representative was manually identified using seqmatch from RDP^58^. Sequences were submitted to European Nucleotide Archive under the accession number PRJEB30105.

### Statistical analysis

The data are presented as means ± standard error of mean (SEM). Statistical analysis was performed using PRISM (version 7.03, GraphPad Software, Inc.). Grubb´s test was used to determine outliers. Bartlett´s test was applied to test homogeneity of variances and data were log-transformed if unequal before performing further statistical tests. A two-factorial analysis of variance (ANOVA) was used to identify statistical differences between groups followed by Tukey’s post hoc test. *P* < 0.05 was defined to be significant. PRIMER-E 7 (Plymouth Marine Laboratory, Plymouth, UK) was used to analyze Illumina amplicon sequencing data^59^. Samples were standardized by total and a similarity matrix was created using Bray-Curtis coefficient^60^. Hierarchical clustering and ordination of the community structures were performed using a Principal Coordinate Analysis (PCoA) plot. For analysis of similarity (ANOSIM), a one-way permutation-based hypothesis testing was used with differences defined as significant if *P* < 0.05. Similarity percentage analysis (SIMPER) was used as detailed by Clarke & Warwick^59^ to calculate the percentage of similarity and dissimilarity and to determine which OTUs contribute to differences found between the diets. Differences in the abundance of OTUs of interest between groups were evaluated using the unpaired Welch t-test.

## Supplementary information

Supplementary Information

## Data Availability

All data generated or analysed during this study are included in this published article (and its Supplementary Information). Raw data are made available upon reasonable request.
