# Supplementary material for: Metformin attenuates the onset of non-alcoholic fatty liver disease and affects intestinal microbiota and barrier in small intestine
Source: Sci Rep. 2019 Apr 30;9:6668. doi: 10.1038/s41598-019-43228-0 (PMC6491483; doi:10.1038/s41598-019-43228-0)
Supplement: Supplementary file 1 — Supplementary Information [file 41598_2019_43228_MOESM1_ESM.pdf]

Metformin attenuates the onset of non-alcoholic fatty liver disease and affects intestinal microbiota and barrier in small intestine

Annette Brandt[1], Angélica Hernández-Arriaga[2], Richard Kehm[3,4], Victor Sánchez[1], Cheng Jun Jin[3], Anika Nier[1], Anja Baumann[1], Amélia Camarinha-Silva[2], Ina Bergheim[1,*]

[1]Department of Nutritional Sciences, R.F. Molecular Nutritional Science, University of Vienna, 1090, Vienna, Austria

[2]Institute of Animal Science, University of Hohenheim, 70599, Stuttgart, Germany

[3]Institute of Nutrition, SD Model Systems of Molecular Nutrition, Friedrich-Schiller-University Jena, 07743 Jena, Germany

[4]Department of Molecular Toxicology, German Institute of Human Nutrition Potsdam-Rehbruecke (DIfE), 14558 Nuthetal, Germany

[*]corresponding author

Supplementary Table S1. Effect of a chronic (6 weeks) and short-term feeding (4 days) of a FFC or control diet ± metformin on markers of inflammation and gluconeogenesis in liver tissue.

	groups				*P*-value		
	**C**	**FFC**	**C+Met**	**FFC+Met**	**DExME**	**ME**	**DE**
**6 weeks**							
***Il10* mRNA**	100±5.6	114±7.7	79.5±8.1	158±12[a,b,c]	<0.05	NS	<0.05
***Clec10a* mRNA**	100±12	80.5±18	101±23	85.9±15	NS	NS	NS
***Pepck* mRNA**	100±18	80.9±26	95.2±16	86.2±23	NS	NS	NS
**4 days**							
***Il10* mRNA**	100±14	140±12	132±34	116±23	NS	NS	NS
***Clec10a* mRNA**	100±18	92.0±6.5	138±32	108±15	NS	NS	NS
***Pepck* mRNA**	100±31	50.4±10[c]	118±20	31.3±4.8[c]	NS	NS	<0.05

Values are means ± standard error of means and are shown as % of control; n=4-8. C: control diet, Clec10a: C-type lectin domain family 10 member A, C+Met: control diet and oral treatment with metformin (300 mg/kg BW/day), DE: diet effect, DExME: interaction between diet and metformin, FFC: fat-, fructose- and cholesterol-rich diet, FFC+Met: fat-, fructose- and cholesterol-rich diet and oral treatment with metformin (300 mg/kg BW/day). ME: metformin effect, Met: metformin, NS: not significant, Il10: interleukin 10, Pepck: phosphoenolpyruvate carboxykinase. [a]*P*<0.05 compared with mice fed a control diet; [b]*P*<0.05 compared with mice fed a FFC diet; [c]*P*<0.05 compared with mice fed a control diet treated with metformin.

Supplementary Table S2. Nutrient composition of control diet and fat-, fructose- and cholesterol-rich diet (FFC).

		Control	FFC
**Crude protein (CP)**	% wt/wt	17.4	16.0
**Crude lipid (CL)**	% wt/wt	5.1	11.8
**Crude fibre**	% wt/wt	5.0	2.0
**Crude ash**	% wt/wt	4.1	4.2
**Corn Starch**	% wt/wt	34.6	5.0
**Sucrose**	% wt/wt	11.0	-
**Glucose**	% wt/wt	-	5.0
**Fructose**	% wt/wt	-	50.0
**Cholesterol**	% wt/wt	-	0.16
**L-Lysine**	% wt/wt	1.43	1.32
**L-Methionine + L-Cystein**	% wt/wt	1.00	0.96
**L-Threonine**	% wt/wt	0.75	0.69
**Calcium**	% wt/wt	0.78	0.77
**Phosphorus**	% wt/wt	0.48	0.47
**Sodium**	% wt/wt	0.23	0.30
**Vitamin A (retinol acetate)**	IU/kg	15000	15000
**Vitamin D_3 (cholecalciferol)**	IU/kg	1500	1500
**Vitamin E (α-tocopherol acetate)**	mg/kg	150	150
**Fatty acids**			
**C4:0**	% wt/wt	-	0.44
**C6:0**	% wt/wt	-	0.29
**C8:0**	% wt/wt	-	0.16
**C10:0**	% wt/wt	-	0.35
**C12:0**	% wt/wt	-	0.40
**C14:0**	% wt/wt	0.02	1.23
**C16:0**	% wt/wt	0.57	3.10
**C18:0**	% wt/wt	0.18	1.14
**C18:1 (n-9)**	% wt/wt	1.28	2.58
**C18:2 (n-6)**	% wt/wt	2.65	0.21
**C18:3 (n-3)**	% wt/wt	0.29	0.06
**Metabolizable energy**	kcal/kg	3752	4254
**CP**	kcal%	19	15
**CL**	kcal%	12	25
**Carbohydrate**	kcal%	69	60

CL: crude lipid, CP: crude protein

Supplementary Table S3. Primer sequences.

	Forward (5´-3´)	Reverse (5´-3´)
**18S**	GTA ACC CGT TGA ACC CCA TT	CCA TCC AAT CGG TAG TAG CG
**Acc**	CTT CCT CCT GAT CAG CAA CTC T	CGT GAG TTT TCC CAA AAT AAG C
**Acox1**	GGG AAT TTG GCA TCG CAG AC	CAT CTC CGT CTG GGC GTA GG
**asma**	CTG ACA GAG GCA CCA CTG AA	CAT CTC CAG AGT CCA GCA CA
**Clec10a**	TGG TCG TCT CCG TGA TTG GA	CCT GCA GTT CCT GCC TGT GA
**Eef2**	GTG ACA GCT GCC TTG CGT GT	GAT GCG CTG GAA GGT CTG GT
**Fasn**	TCT GGG CCA ACC TCA TTG GT	GAA GCT GGG GGT CCA TTG TG
**Il10**	CAG GGC CCT TTG CTA TGG TG	CGG CTG GGG GAT GAC AGT AG
**Il1b**	TGG CTG TGG AGA AGC TGT GG	GTC CGA CAG CAC GAG GCT TT
**Mmp13**	AGA AGT GTG ACC CAG CCC TA	GCG CAA GAA GAA TCT GTC TTT
**Occ**	CAT CAG CCA TGT CCG TGA GG	GGG GCG ACG TCC ATT TGT AG
**Pepck**	CCC TGG GAG ATG GGG AGT TC	CCC ACC ATA TCC GCT TCC AA
**Srebp1c**	ACC GGC TAC TGC TGG ACT GC	AGA GCA AGA GGG TGC CAT CG
**Tlr4**	AGC CAT TGC TGC CAA CAT CA	GCT GCC TCA GCA GGG ACT TC
**Vimentin**	GCC GCA GCC TCT ATT CCT CA	CTC GAG GAA GAG CAC CTT GT
**Zo1**	GCA GAC TTC TGG AGG TTT CG	CTT GCC AAC TTT TCT CTG GC

Acc: acetyl coA carboxylase, Acox1: acyl coA oxidase-1, asma: α-smooth muscle actin, Clec10a: C-type lectin domain family 10 member A, Eef2: eukaryotic elongation factor 2, Fasn: fatty acid synthase, Il10: interleukin 10, Il1b: interleukin 1β, Mmp: matrix-metalloproteinase, Occ: occludin, Pepck: phosphoenolpyruvate carboxykinase, Srebp1c: sterol regulatory element-binding protein 1, Tlr: toll-like receptor, Zo1: zonula occludens-1

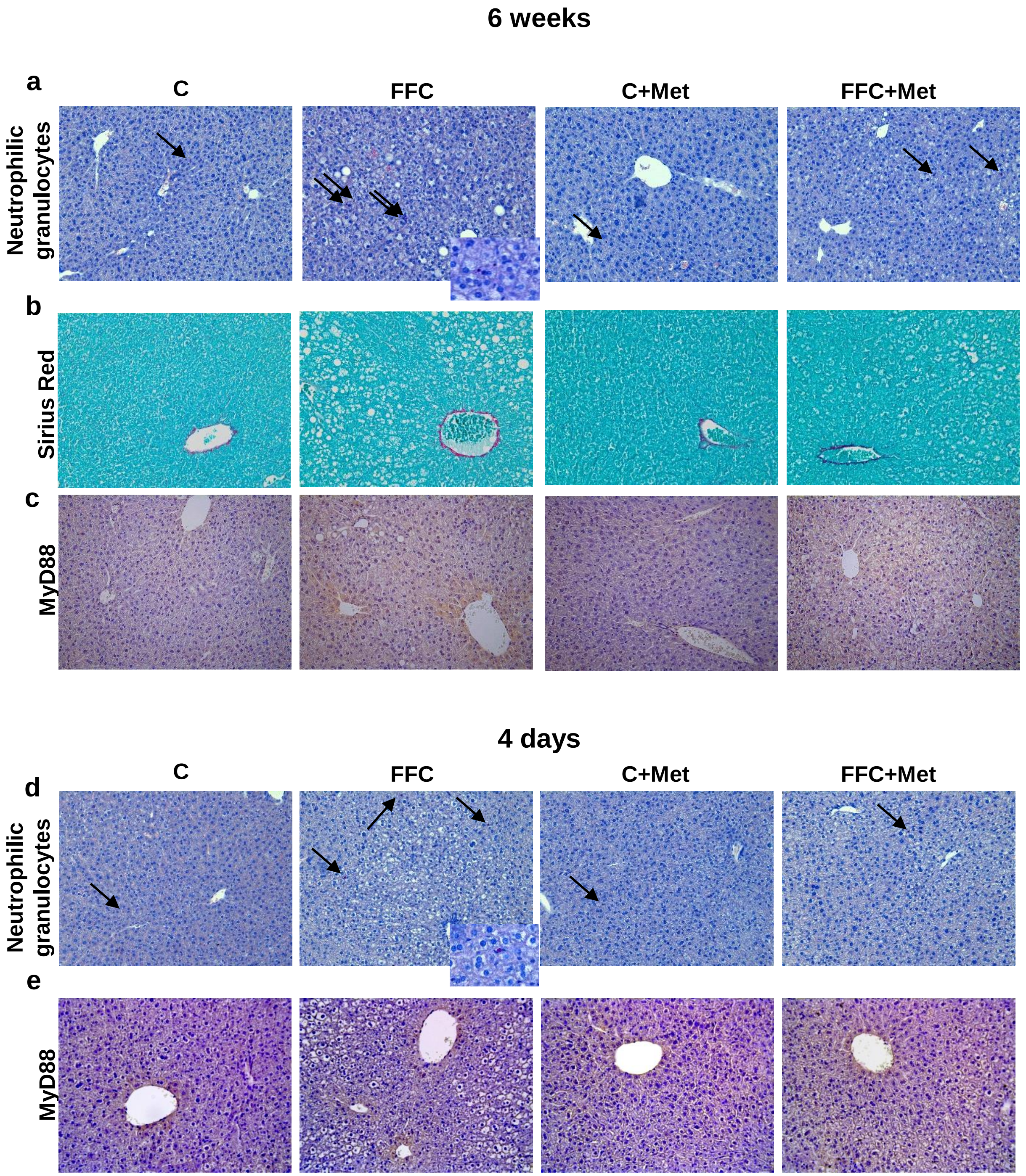

Supplementary Figure S1: Representative pictures of neutrophilic granulocytes, Sirius Red staining and MyD88 protein staining in long-term feeding (6 weeks) and short-term feeding (4 days) of a FFC or control diet ± metformin. (a,d) Neutrophilc granulocytes in liver sections (200x; arrows are indicators for neutrophilic granulocytes; insertion in FFC sections 630x), (b) Sirius red staining in liver section and (c, e) MyD88 in liver tissue (200x) in long-term feeding and short-term feeding. C: control diet; C+Met: control diet and oral treatment with metformin (300 mg/kg BW/day); FFC: fat-, fructose- and cholesterol-rich diet; FFC+Met: fat-, fructose- and cholesterol-rich diet and oral treatment with metformin (300 mg/kg BW/day); MyD88: myeloid differentiation primary response 88.

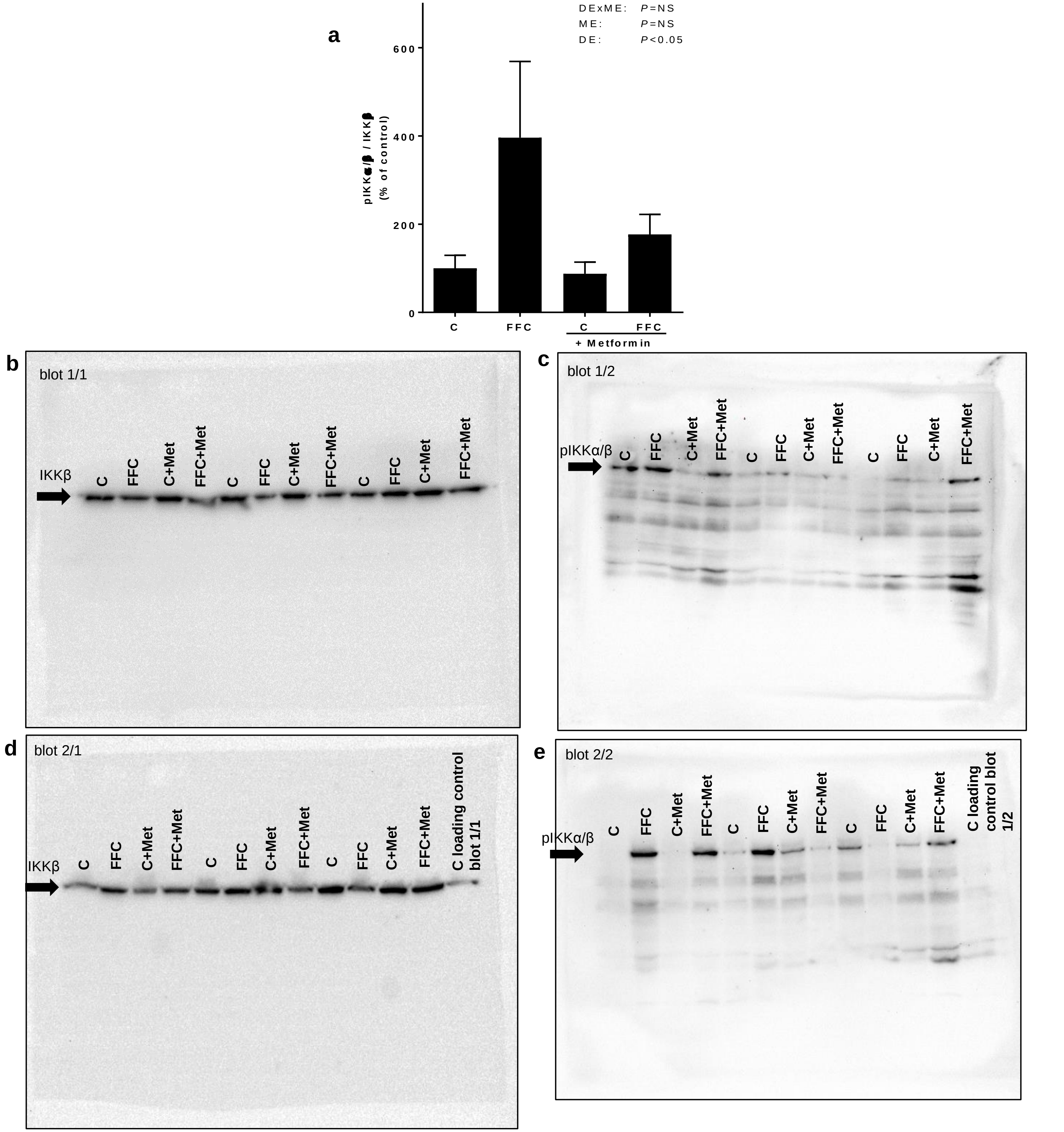

Supplementary Figure S2: Effect of a long-term feeding (6 weeks) of a FFC or control diet ± metformin on phosphorylation status of inhibitor of nuclear factor kappa-B kinase subunit α and β (pIKKα/β) in liver tissue. (a) Densitometric analysis of pIKKα/β and IKKβ Western blot and full length blot of (b,d) IKKβ and (c,e) pIKKα/β; n=5-6; samples derive from the same experiment and blots were processed in parallel. Data presented as means ± standard error of means. C: control diet; C+Met: control diet and oral treatment with metformin (300 mg/kg BW/day); DE: diet effect; DExME: interaction between diet and metformin; FFC: fat-, fructose- and cholesterol-rich diet; FFC+Met: fat-, fructose- and cholesterol-rich diet and oral treatment with metformin (300 mg/kg BW/day); ME: metformin effect ; NS: not significant.

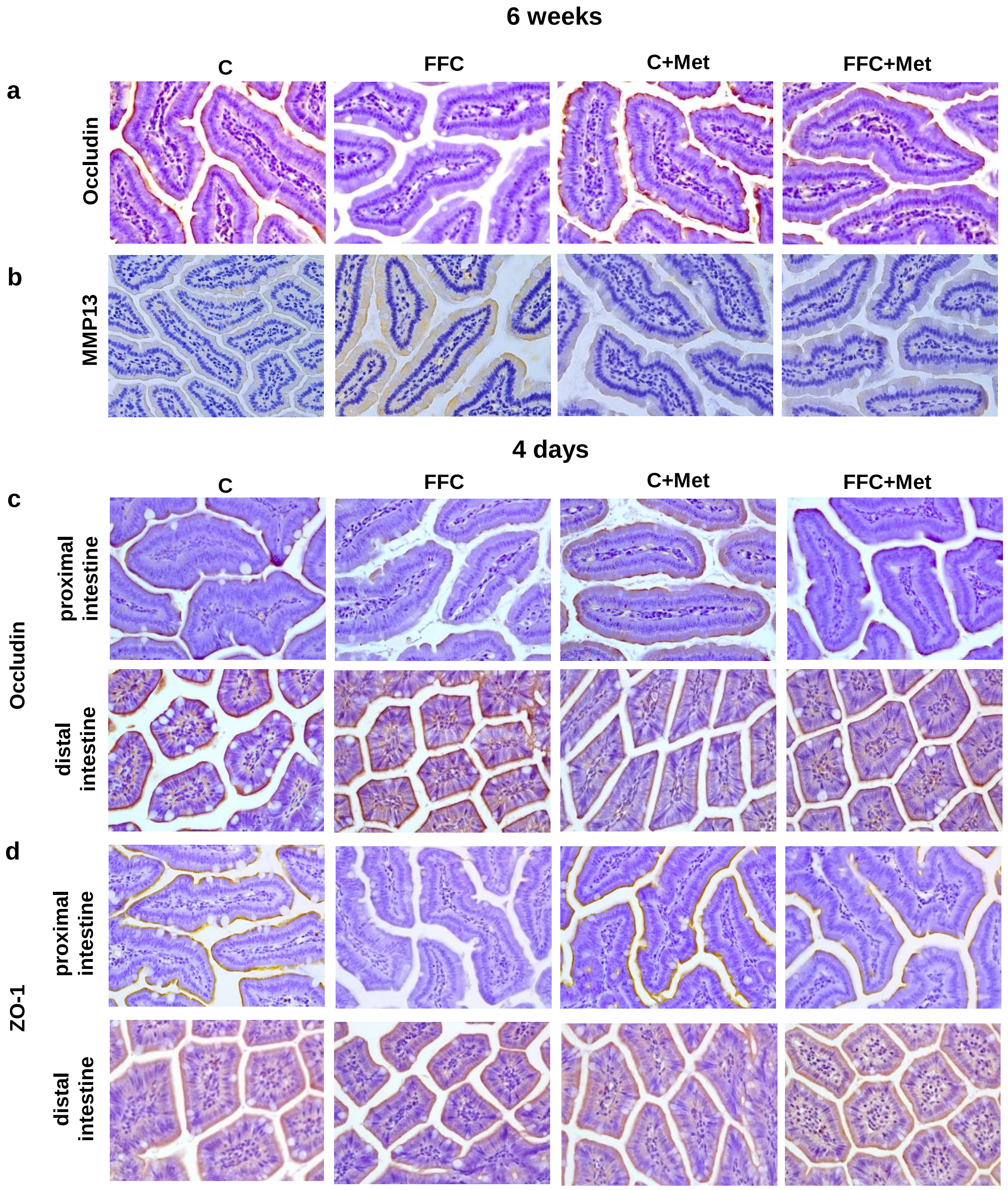

Supplementary Figure S3: Representative pictures of immunohistochemical stainings in a long-term feeding (6 weeks) and short-term feeding (4 days) of a FFC or control diet ± metformin. (a) Occludin (400x) and (b) MMP13 (400x) protein staining in proximal intestinal tissue in long-term trial as well as (c) occludin (400x) and (d) ZO-1 (400x) in proximal and distal intestinal tissue in short-term trial. C: control diet; C+Met: control diet and oral treatment with metformin (300 mg/kg BW/day); FFC: fat-, fructose- and cholesterol-rich diet; FFC+Met: fat-, fructose- and cholesterol-rich diet and oral treatment with metformin (300 mg/kg BW/day); MMP13: matrix-metalloproteinase 13; ZO-1: zonula occludens-1.

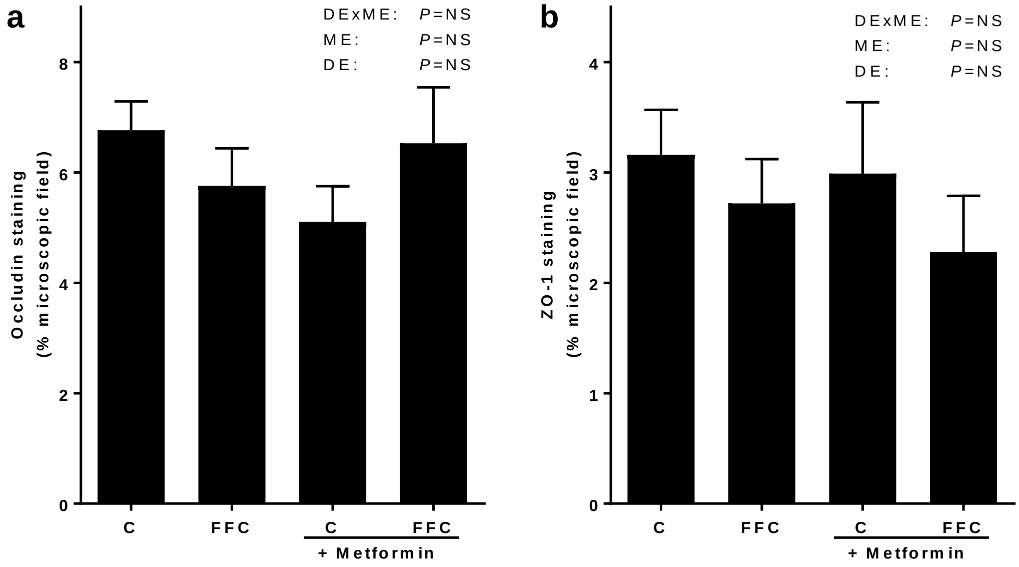

Supplementary Figure S4: Effect of a short-term feeding (4 days) of a FFC or control diet ± metformin on tight junction proteins in distal small intestine. Densitometric analysis of (a) occludin and (b) ZO-1 protein staining in tissue of distal small intestine; n=7-8. Data presented as means ± standard error of means. C: control diet; C+Met: control diet and oral treatment with metformin (300 mg/kg BW/day); DE: diet effect; DExME: interaction between diet and metformin; FFC: fat-, fructose- and cholesterol-rich diet; FFC+Met: fat-, fructose- and cholesterol-rich diet and oral treatment with metformin (300 mg/kg BW/day); ME: metformin effect ; NS: not significant; ZO-1: zonula occludens-1.

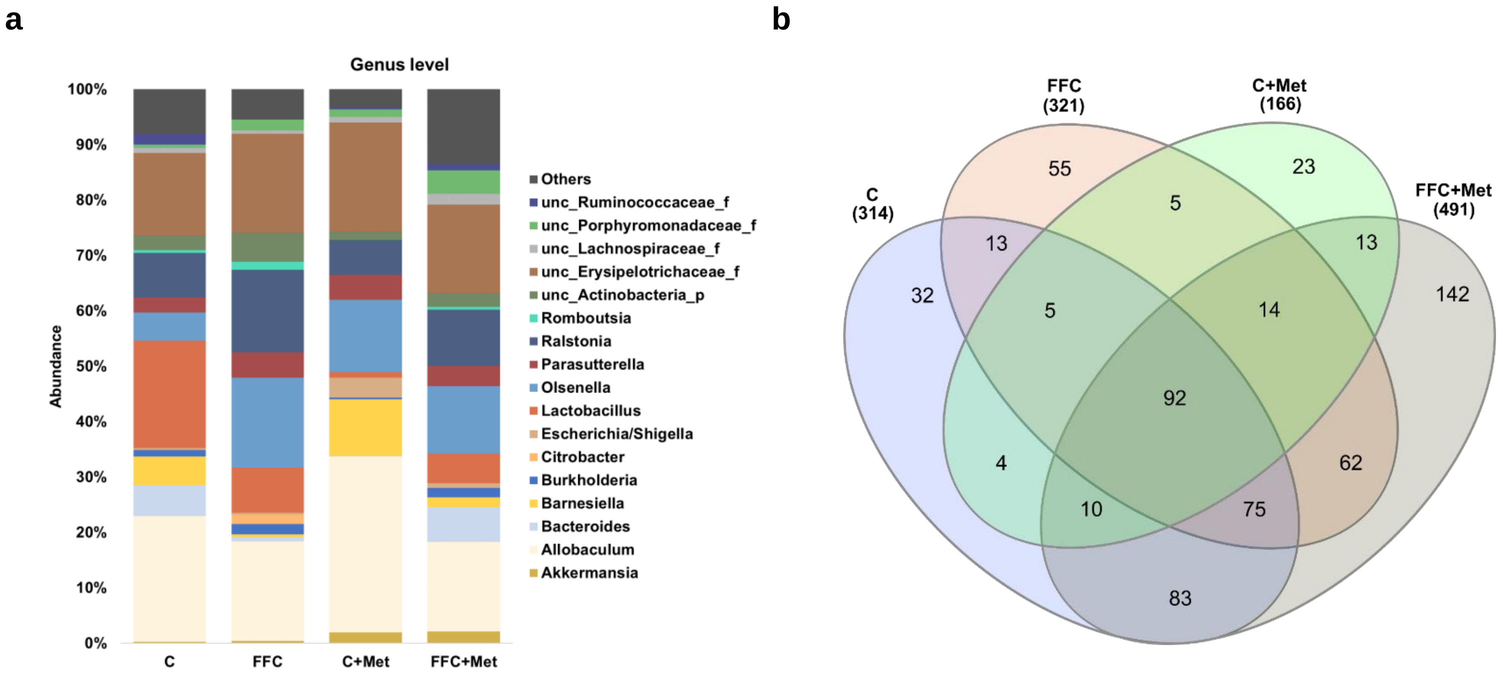

Supplementary Figure S5: Effect of a short-term feeding (4 days) of a FFC or control diet ± metformin on microbial community in small intestine. (a) At genus level. (b) Venn diagram with OTUs present in the samples of each diet; n=4-6. C: control diet; C+Met: control diet and oral treatment with metformin (300 mg/kg BW/day); FFC: fat-, fructose- and cholesterol-rich diet; FFC+Met: fat-, fructose- and cholesterol-rich diet and oral treatment with metformin (300 mg/kg BW/day); unc: unclassified.

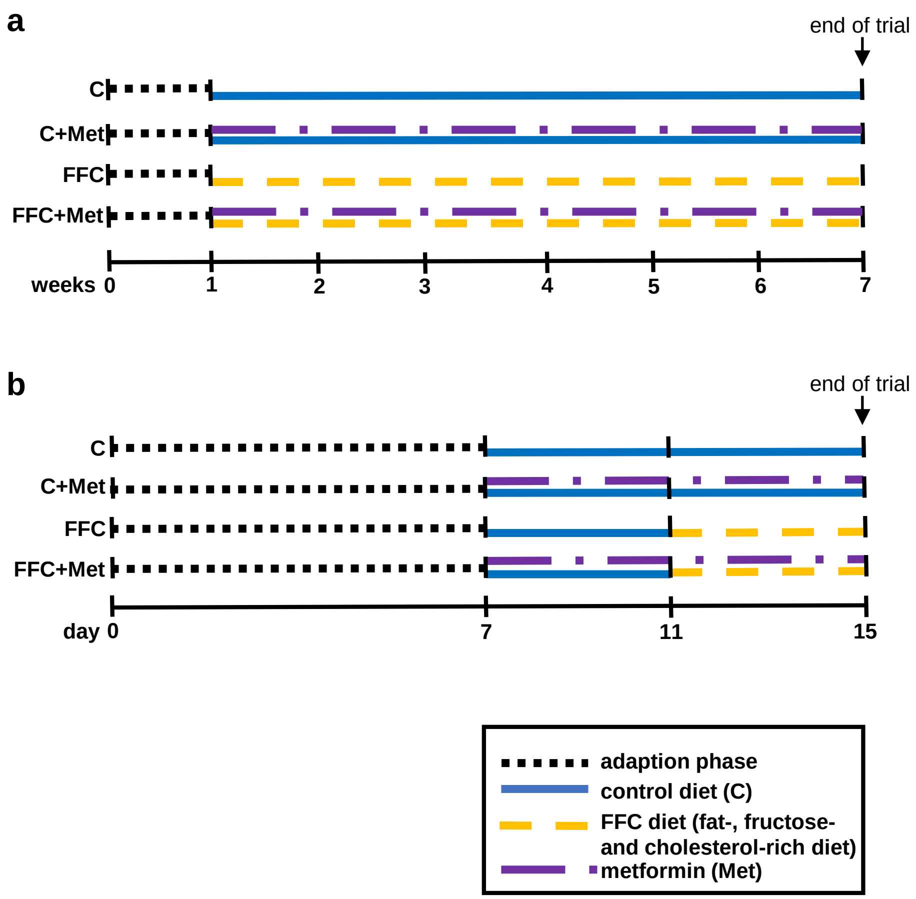

Supplementary Figure S6: Summary of study design. (a) Chronic feeding trial over 6 weeks and (b) short-term feeding trial fed the FFC or control diet over 4 days ± metformin. C: control diet; C+Met: control diet and oral treatment with metformin (300 mg/kg BW/day); FFC: fat-, fructose- and cholesterol-rich diet; FFC+Metformin: fat-, fructose- and cholesterol-rich diet and oral treatment with metformin (300 mg/kg BW/day).
